# Crystal structures of the selenoprotein glutathione peroxidase 4 in its apo form and in complex with the covalently bound inhibitor ML162

**DOI:** 10.1107/S2059798320016125

**Published:** 2021-01-26

**Authors:** Dieter Moosmayer, André Hilpmann, Jutta Hoffmann, Lennart Schnirch, Katja Zimmermann, Volker Badock, Laura Furst, John K. Eaton, Vasanthi S. Viswanathan, Stuart L. Schreiber, Stefan Gradl, Roman C. Hillig

**Affiliations:** a Research and Development, Pharmaceuticals, Bayer AG, 13353 Berlin, Germany; b Broad Institute, Cambridge, Massachusetts, USA

**Keywords:** glutathione peroxidase 4, anti-oxidative defense system, covalent inhibitors, oxidoreductases, ferroptosis, GPX4, ML162

## Abstract

Wild-type human glutathione peroxidase 4 (GPX4) was co-expressed with SBP2 (selenocysteine insertion sequence-binding protein 2) in human HEK cells to achieve efficient production of this selenocysteine-containing enzyme on a preparative scale for structural biology. The protein was purified and crystallized, and the crystal structure of the wild-type form of GPX4 was determined at 1.0 Å resolution. The overall fold and the active site are conserved compared with previously determined crystal structures of mutated forms of GPX4. A mass-spectrometry-based approach was developed to monitor the reaction of the active-site selenocysteine Sec46 with covalent inhibitors. This, together with the introduction of a surface mutant (Cys66Ser), enabled the crystal structure determination of GPX4 in complex with the covalent inhibitor ML162 [(*S*)-enantiomer]. The mass-spectrometry-based approach described here opens the path to further co-complex crystal structures of this potential cancer drug target in complex with covalent inhibitors.

## Abstract 2

The crystal structure of the human selenocysteine-containing protein glutathione peroxidase 4 (GPX4) was determined at 1.0 Å resolution. A mass-spectrometry-based approach was developed to monitor the formation of adducts of the active-site selenocysteine Sec46 with covalent inhibitors. The crystal structure of Sec46-containing GPX4 in complex with the covalent inhibitor ML162 [(*S*)-enantiomer] was determined at 1.54 Å resolution.

## 1. Introduction   

The glutathione peroxidases (GPXs) are part of the cellular antioxidative defense system (Brigelius-Flohé & Maiorino, 2013 ▸). They catalyze the reduction of hydrogen peroxide or organic hydroperoxides to water or the corresponding alcohols, respectively, typically using glutathione as a reductant. The isoform GPX4 belongs to a subfamily of selenium-containing GPX enzymes and is unique in that it can also reduce hydroperoxides in complex lipids such as phospho­lipids, cholesterol and cholesteryl ester hydroperoxides, even when they are inserted into biomembranes or lipoproteins (Thomas *et al.*, 1990 ▸; Kühn & Borchert, 2002 ▸). Unusually for a GPX enzyme, GPX4 can use not only glutathione but also other thiol-containing proteins as reductants (Godeas *et al.*, 1996 ▸; Maiorino *et al.*, 2005 ▸).

Three different isoforms have been reported for GPX4, a cytosolic GPX4 (cGPX4), a mitochondrial GPX4 (mGPX4) and sperm nuclear GPX4 (snGPX4), all of which are splice variants derived from the same gene (Brigelius-Flohé & Maiorino, 2013 ▸). The cytosolic isoform of human GPX4 (UniProt identifier P36969-2; referred to as GPX4 in the following text) is a 19.5 kDa protein which comprises 170 residues and features a selenocysteine (Sec, U) at position 46. The catalytic mechanism of the GPX reaction involves redox shuttling of this selenocysteine between the redox states selenol (Se–H) and selenenic acid (Se–OH) (Flohé, 1988 ▸; Borchert *et al.*, 2018 ▸; Brigelius-Flohé & Maiorino, 2013 ▸). The catalytic site of GPX enzymes was initially suggested to consist of a catalytic triad formed by a selenocysteine, a glutamine and a tryptophan residue (Epp *et al.*, 1983 ▸). This was later extended by a conserved asparagine to form a catalytic tetrad (Tosatto *et al.*, 2008 ▸; Maiorino *et al.*, 1995 ▸), which in human GPX4 consists of Sec46, Gln81, Trp136 and Asn137 (Borchert *et al.*, 2018 ▸; Tosatto *et al.*, 2008 ▸).

**Figure 1:**
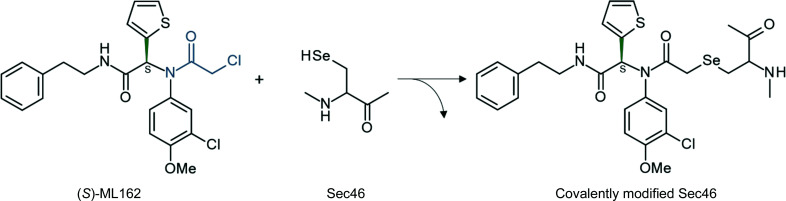
The small-molecule inhibitor ML162 targeting Sec46 of human GPX4. The (*S*)-enantiomer (stereocenter depicted in green, covalent warhead α-­chloroacetamide in blue) is shown. The postulated product of the covalent reaction with Sec46 of GPX4^WT^ is shown on the right.

Recently, GPX4 has been identified as a key player in ferroptosis, a non-apoptotic cell death resulting from the iron-dependent accumulation of lipid reactive-oxygen species (Yang *et al.*, 2014 ▸; Dixon *et al.*, 2012 ▸). Yang and coworkers discovered that ferroptosis can be induced by two different classes of small molecules that either deplete glutathione, thereby inactivating GPX4, or inhibit GPX4 directly. Of particular interest is that cancer cells in a therapy-induced and therapy-resistant persister state which is thought to underlie cancer relapse are particularly dependent on GPX4 to prevent them from undergoing ferroptotic cell death (Viswanathan *et al.*, 2017 ▸; Hangauer *et al.*, 2017 ▸). GPX4 has thus arisen as a potential cancer drug target, and several small molecules that were originally identified as modulators of ferroptosis in cancer cells have now been recognized as inhibitors of GPX4 and may serve as starting points towards future small-molecule drugs for GPX4, including RSL3 (Yang & Stockwell, 2008 ▸), ML162 and ML210 (Weïwer *et al.*, 2012 ▸) (Fig. 1 ▸).

All known GPX4 inhibitors with cellular activity inhibit GPX4 covalently via reaction with the selenocysteine residue (Eaton *et al.*, 2020 ▸; Yang *et al.*, 2014 ▸, 2016 ▸). Cellular (Yang *et al.*, 2014 ▸) and biochemical (Sakamoto *et al.*, 2017 ▸) screens to identify GPX4 inhibitors have so far uncovered two classes of covalent inhibitors: activated alkyl chlorides (for example RSL3 and ML162) and masked nitrile oxides [for example ML210 (Eaton *et al.*, 2020 ▸) and diacylfuroxans (Eaton *et al.*, 2019 ▸)]. These electrophiles present limitations regarding drug development. However, recent success in covalent inhibitor development has led to a revival of interest in this class of drugs (Baillie, 2016 ▸). In particular, acrylamide-based inhibitors of epidermal growth factor receptor (for example afatinib and neratinib) and Bruton’s tyrosine kinase (ibrutinib) have led to FDA-approved therapeutics (Baillie, 2016 ▸) or show promise in addressing difficult-to-drug targets such as KRAS^G12C^ (Goody *et al.*, 2019 ▸; Nagasaka *et al.*, 2020 ▸). Understanding the structural basis of GPX4 inhibitor binding may enable the development of improved GPX4-targeting compounds with drug-like properties.

The production of selenocysteine-containing proteins in sufficient quantities for structural analysis is challenging. The first crystal structures of human GPX4 were therefore solved using the U46C mutant (PDB entry 2obi; Scheerer *et al.*, 2007 ▸) and the U46G mutant (PDB entry 2gs3; Structural Genomics Consortium, unpublished work) of GPX4. Similarly, the first crystal structures of GPX4 in complex with noncovalent cyclic peptide inhibitors were solved using a construct in which the active-site selenocysteine residue was mutated to cysteine and six further cysteine residues were mutated to noncysteine residues (Sakamoto *et al.*, 2017 ▸). Recently, Borchert and coworkers succeeded in solving the first crystal structure of a Sec46-containing variant of GPX4, where again all of the remaining cysteine residues of GPX4 were mutated to non­cysteine residues (Borchert *et al.*, 2018 ▸). In order to produce the true wild-type protein, we employed a novel approach, first described by Eaton *et al.* (2020 ▸), in which wild-type GPX4 was co-expressed with selenocysteine insertion sequence-binding protein 2 (SBP2) in mammalian cells on a 10–30 l scale. This enabled the crystallization of wild-type GPX4 and the development of a mass-spectrometry-based approach to produce GPX4 homogenously modified with covalent inhibitors targeting Sec46, resulting in the crystal structure of GPX4 in complex with the covalent inhibitor ML162 (Weïwer *et al.*, 2012 ▸; Yang *et al.*, 2014 ▸, 2016 ▸).

## 2. Materials and methods   

### 2.1. Plasmids, mammalian cell expression and purification of recombinant GPX4 proteins   

All GPX4 expression cassettes used in this work are based on DNA sequence X71973 and encode the amino-acid sequence Met1–Phe170 (UniProt accession code P36969-2; cytosolic isoform, wild type and with a C66S mutation, as indicated below). These cassettes encode a 5′-end PstI site, a Kozak sequence preceding the start codon Met1, the GPX4 protein, a C-terminal His_6_ tag followed by a stop codon, a SECIS (selenocysteine insertion sequence) element and a BclI 3′-end. Four different SECIS elements were tested in the expression cassette: a previously described chimeric element (Novoselov *et al.*, 2007 ▸), the human element from GPX4 (X71973.1; base pairs 675–863), the element from human selenoprotein N (SelN; NM_206926.1; base pairs 2802–2904) and the element from *Toxoplasma gondii* (Toxopl; AK318349.1; base pairs 1067–1305). For the final preparative-scale experiments the GPX4 cassettes [wild type (WT) as well as with a C66S mutation] were used with the chimeric SECIS. Additionally, a DNA cassette encoding full-length rat SBP2 protein (Q9QX72) with a Kozak sequence and EcoRI and BamHI sites in the 5′ and 3′ regions, respectively, was applied. All DNA cassettes were synthesized by GeneArt Technology at Life Technologies and were subcloned into the mammalian expression vector pTT5 (Durocher *et al.*, 2002 ▸) via the mentioned restriction sites. The DNA constructs and the proteins that they encode are designated GPX4_WT_His_6_, GPX4_C66S_His_6_ and SBP2. The final expression vectors are denoted with the suffix ‘-pTT5’. Expression plasmids encoding GPX4-pTT5 and SBP2-pTT5 were amplified in *Escherichia coli* (One Shot TOP10) and were purified using a QIAprep Minispin Kit (Qiagen; catalog No. 27104; for small scale) and NucleoBond PC 10000 EF (Macherey-Nagel; catalog No. 740548) to yield highly pure plasmid preparations that are suitable for transfection.

For protein expression, the GPX4-pTT5 plasmid together with SBP2-pTT5 was transfected transiently in HEK293-6E cells. Transfection mixtures were prepared by combining a total of 1 µg plasmid DNA [comprising both GPX4-pTT5 and SBP2-pTT5 plasmids in a 4:1(*w*:*w*) plasmid ratio] for each 1 ml of transfected cell culture with PEI transfection reagent (polyethylenimine, linear; Polysciences; catalog No. 23966) at a ratio of 1:2(*w*:*w*). This mixture was then added to F17 medium (without any supplements), mixed carefully and incubated for 15 min at room temperature. It was then added to HEK293-6E cells cultured at a density of 1.6 × 10^6^ cells ml^−1^ in F17 Medium (Gibco, Invitrogen; catalog No. 05-0092DK) supplemented with 0.1% Pluronic F68 (Gibco; catalog No. 24040), 200 m*M* l-alanylglutamine (Gluta-Max, Invitrogen; catalog No. 25030) and 25 µg ml^−1^ G418 (PAA; catalog No. P02-012). 5 h post-transfection, 1 µ*M* sodium selenite was added and the culture was shaken for 72 h at 310 K in culture vessels ranging in volume from 2 ml to a 10 l bioreactor (Cultibag RM, Sartorius Stedim Biotech). The cells were then harvested by centrifugation (30 min, 1000*g*, 288 K) and the resulting cell pellets were stored at 193 K.

The purification of recombinant GPX4 proteins expressed in HEK293-6E cells was performed in two steps using an ÄKTA avant chromatography system. An initial affinity-chromatography step (immobilized metal ion-affinity chromatography; IMAC) was followed by a size-exclusion chromatography (SEC) step (Superdex 75, GE). The pellet from the transfected cells was suspended in lysis buffer (50 m*M* Tris–HCl pH 7.4, 300 m*M* NaCl, 10 m*M* imidazole, 0.1% NP40, 1 m*M* DTT, cOmplete EDTA-free protease-inhibitor cocktail). The supernatant of the lysate was applied onto an Ni–NTA column (Macherey-Nagel; catalog No. 745400) and washed with buffer (50 m*M* Tris–HCl pH 7.4, 300 m*M* NaCl, 10 m*M* imidazole, 1 m*M* dithiothreitol). Bound protein was eluted from the column using the same buffer supplemented with 300 m*M* imidazole. Elution fractions from affinity chromatography were concentrated using Amicon Ultra 15 centrifugal filters (10 kDa molecular-weight cutoff; Millipore; catalog No. UFC901024) and subjected to SEC. The resulting peak fractions were collected, pooled and concentrated again. The buffer used for SEC and final sample preparation was 50 m*M* Tris–HCl pH 8.0, 150 m*M* NaCl, 5 m*M* tris(2-carboxyethyl)phosphine (TCEP). The final concentration of the purified GPX4 was typically about 1.5 mg ml^−1^, and the yield after final purification was approximately 1 mg of product per litre of culture. The final sample was concentrated to 18 mg ml^−1^ and stored at 193 K until use. GPX4^C66S^ was expressed and purified as described above for GPX4^WT^, concentrated to 14–16 mg ml^−1^ and stored at 193 K.

### 2.2. Synthesis of ML162   

The small-molecule inhibitor ML162 {2-chloro-*N*-(3-chloro-4-methoxyphenyl)-*N*-[2-oxo-2-(phenethylamino)-1-(thiophen-2-yl)ethyl]acetamide} was synthesized as a racemate as described previously (Weïwer *et al.*, 2012 ▸). The pure enantiomers were separated by chiral supercritical fluid chromatography (SFC). For an analytical approach, an Agilent 1260 SCF instrument was used (with an Aurora SFC module; column, Chiralpak IA 5 µm 100 × 4.6 mm; eluent A, CO_2_; eluent B, ethanol; isocratic 20% B; flow rate, 4 ml min^−1^; temperature, 313 K; BPR, 150 bar; UV, 220 nm). The two enantiomers eluted with retention times of 1.62 min (peak 1) and 4.31 min (peak 2). For preparative production, 25 mg of the racemate was used. The instrument used was a Sepiatec Prep SFC100 (column, Chiralpak IA 5 µm 250 × 30 mm; eluent A, CO_2_; eluent B, ethanol; isocratic 20% B; flow rate, 100 ml min^−1^; temperature, 313 K; BPR, 150 bar; UV, 220 nm). The (*S*)-enantiomer eluted with a retention time of 6.0–10.0 min, a yield of 7 mg, 99.2% enantiomeric excess and >90% purity. The (*R*)-enantiomer eluted with a retention time of 16.0–21.0 min, a yield of 7 mg, 99.2% enantiomeric excess and >95% purity. Peak 1 was assigned as the (*S*)-enantiomer based on the co-crystal structure with GPX4^C66S^ reported below.

### 2.3. Crystallization of apo wild-type GPX4   

Wild-type GPX4 (GPX4^WT^) was crystallized by vapor diffusion using the hanging-drop method. Rod-shaped crystals appeared within hours at 293 K in drops consisting of 1 µl protein solution (17.6 mg ml^−1^ in 50 m*M* Tris–HCl pH 8.0, 150 m*M* NaCl, 5 m*M* TCEP) and 1 µl reservoir solution [18–21%(*w*/*v*) PEG 3350, 0.1 *M* MES pH 6.0, 5%(*v*/*v*) ethanol] and reached their final size after one day.

### 2.4. Data collection, processing, structure solution and refinement of apo wild-type GPX4   

A GPX4^WT^ crystal was briefly immersed in cryobuffer (reservoir supplemented with 15% glycerol) and flash-cooled in liquid nitrogen. Data were collected at 100 K on beamline 14.1 at the Helmholtz-Zentrum Berlin (HZB; wavelength 0.9184 Å) using a PILATUS detector. Data were processed using *XDS* (Kabsch, 2010 ▸) and *XDSAPP* (Sparta *et al.*, 2016 ▸). Data-collection statistics are listed in Table 1 ▸. The crystal diffracted to a resolution of 1.0 Å and belonged to space group *P*1, with one GPX4 molecule per asymmetric unit. The structure was solved by molecular replacement using *Phaser* (McCoy *et al.*, 2007 ▸) from the *CCP*4 suite (Winn *et al.*, 2011 ▸) with PDB entry 2obi (Scheerer *et al.*, 2007 ▸) as a search model and was refined using *REFMAC*5 (Murshudov *et al.*, 2011 ▸). The initial model was rebuilt using *Coot* (Emsley *et al.*, 2010 ▸) followed by several cycles of refinement and rebuilding. The final refinement statistics are summarized in Table 2 ▸.

### 2.5. Small-scale mass-spectrometry (MS) time-course experiments   

For GPX4^WT^ treated with the racemic mixture of ML162, different molar ratios of protein (in 50 m*M* Tris–HCl pH 8.0, 150 m*M* NaCl, 5 m*M* TCEP) and ligand were incubated at two different protein concentrations for up to 4 h (total volume of 15 µl per reaction). The protein concentration was adjusted to 10 and 100 µ*M*, respectively, and the ligand was added in a fivefold, tenfold and 20-fold molar excess. The reaction was quenched by adding 1 µl 5%(*v*/*v*) trifluorocacetic acid (TFA) to a 15 µl reaction volume for LC-MS analysis.

The extent of covalent binding was assessed by LC-MS analysis using a Waters SYNAPT G2-S quadrupole time-of-flight mass spectrometer connected to a Waters nanoAcquity UPLC system. Samples were loaded onto a 2.1 × 5 mm MassPrep C4 guard column (Waters) and desalted with a short gradient (3 min) of increasing acetonitrile concentration at a flow rate of 100 µl min^−1^. The spectra were analyzed using *MassLynx* version 4.1 and deconvoluted with the *MaxEnt*1 algorithm. Percent binding was determined using *BiopharmaLynx* (Waters).

For the GPX4^C66S^ mutant, a similar time-course experiment was carried out to follow the covalent reaction process. Here, the protein was tested at 50 µ*M* with a 2.5-fold, fivefold and tenfold molar excess of the ligand and at 100 µ*M* with a fivefold and tenfold molar excess.

### 2.6. Covalent modification, crystallization and structure determination of GPX4^WT^ with ML162 (racemate)   

For the preparation of GPX4^WT^ covalently modified with the racemate of ML162, two batches were purified. 5 mg GPX4^WT^ (50 µ*M* final concentration) was incubated either for 35 min with a fivefold molar excess of ML162 (approach 1) or for 4 h with a 50-fold molar excess of ML162 (approach 2). The reaction mixtures were centrifuged (3 min, 3220*g*) and subjected to SEC (Superdex 75). The peak fractions were concentrated to 16 mg ml^−1^ and sitting drops were pipetted using a Mosquito robot (0.2 µl protein solution and 0.2 µl reservoir) and stored at 293 K. Crystals were only identified from approach 1 and grew in drops containing 20%(*w*/*v*) PEG 3350, 200 m*M* magnesium formate. They were cryoprotected using reservoir solution supplemented with 15% glycerol. A data set was collected to 2.3 Å resolution on beamline P11 at PETRA III, DESY, Hamburg (wavelength 1.0332 Å) using a PILATUS3 6M detector. The crystal belonged to space group *P*2_1_ and diffracted to a resolution of 2.3 Å. The structure was solved by molecular replacement (with *Phaser*) using the apo structure described here as a search model, followed by refinement using *REFMAC*5 (Murshudov *et al.*, 2011 ▸) and *Coot* (Emsley *et al.*, 2010 ▸). The structure contains two GPX4 molecules in the asymmetric unit. Chain *B* showed extra density at Sec46 and on Cys66 which was interpreted as covalently bound ML162. However, the density at both residues was too weak to place the ligand unambiguously. Work on this structure was therefore terminated.

### 2.7. Covalent modification, crystallization and structure determination of GPX4^C66S^ with (*S*)-ML162   

For preparation of GPX4^C66S^-His_6_ covalently modified with (*S*)-ML162, the protein (50 µ*M*) was incubated with 125 µ*M* inhibitor for 30 min at 293 K and then centrifuged (3 min, 3220*g*). The reaction was stopped by SEC (Superdex 75). Peak fractions were concentrated to 13.5 mg ml^−1^ and sitting drops were pipetted using a Mosquito robot (0.2 µl protein solution and 0.2 µl reservoir solution, 293 K). Crystals grew within one day with 0.2 *M* ammonium sulfate, 20%(*w*/*v*) PEG 3350 as the reservoir solution. They were briefly immersed in reservoir solution supplemented with 15% glycerol and flash-cooled in liquid nitrogen. A single crystal was mounted at 100 K on a Rigaku MicroMax-007 HF diffractometer (wavelength 1.54 Å) equipped with a PILATUS 200K detector. Data collection and processing was carried out using *HKL*-3000 (Minor *et al.*, 2006 ▸). The structure was solved by molecular replacement with *Phaser* using the apo structure described above as a search model and was rebuilt and refined using *Coot* and *REFMAC*5. The crystal belonged to space group *P*2_1_2_1_2_1_ and diffracted to a resolution of 1.54 Å. The structure contains a single GPX4^C66S^ molecule in the asymmetric unit. Clear difference density allowed the building of the complete inhibitor (*S*)-ML162 covalently linked to Sec46. For parameterization, a 3D model of (*S*)-ML162 was generated using *Discovery Studio* (Dassault Systèmes BIOVIA) and parameter files were generated using *PRODRG* (Schüttelkopf & van Aalten, 2004 ▸). The final data-collection and refinement statistics are summarized in Tables 1 ▸ and 2 ▸, respectively.

## 3. Results and discussion   

**Figure 2:**
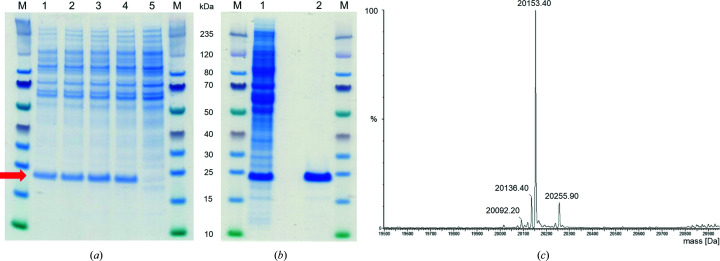
(*a*) Comparison of the effect of different SECIS elements on GPX4 expression when co-expressed with rat SBP2 using transient transfection in HEK293-6E cells. Transfected cells were lysed and GPX4 was partially purified by IMAC, and the eluates were analyzed by SDS–PAGE. Lanes 1–4 show expression of GPX4 with the chimeric SECIS (lane 1), with SelN SECIS (lane 2), with GPX4 SECIS (lane 3) and with *T. gondii* SECIS (lane 4). Lane 5 shows Mock-transfected HEK293-6E cells. The arrow indicates the band representing GPX4^WT^ (molecular weight 21 155 Da). (*b*) Purification steps for GPX4^WT^ analyzed by SDS–PAGE. Lane 1 shows the pooled fractions after IMAC as subjected to gel filtration; lane 2 shows the pooled fractions of the final gel-filtration step. (*c*) Mass-spectrometric analysis of purified GPX4^WT^ is consistent with 100% incorporation of one Se atom. The deconvoluted spectrum is shown.

The wild-type form of the selenocysteine-containing protein GPX4 with a C-terminal His_6_ tag (GPX4^WT^) was expressed and purified following the protocol described previously for GPX4 with a FLAG-tag (Eaton *et al.*, 2020 ▸). In brief, full-length GPX4 (cytosolic isoform; residues 1–170) with a His_6_ tag directly fused to the C-terminus was co-expressed with SECIS-binding protein 2 (SBP2) in HEK293-6E cells. Four different SECIS elements were tested in the expression cassette: a previously described chimeric element (Novoselov *et al.*, 2007 ▸), the human element from GPX4 (X71973-1), the element from SelN (NM_206926-1) and that from *T. gondii* (AK318349.1). All were found to be equally efficient in the expression of GPX4^WT^ when co-expressed with SBP2 (SECIS-binding protein 2) in HEK293-6E cells (Fig. 2 ▸ *a*). The protein was purified via an affinity-chromatography step followed by a size-exclusion chromatography step (Fig. 2 ▸ *b*). Mass-spectrometric analysis of purified GPX4^WT^ (Fig. 2 ▸ *c*) confirmed the presence of selenocysteine and revealed that the N-terminal residues Met1 and Cys2 were missing and that the N-terminus was acetylated (expected molecular weight 20 155 Da, measured molecular weight 20 153 Da).

### 3.1. Comparison of the wild-type and mutant crystal structures   

**Figure 3:**
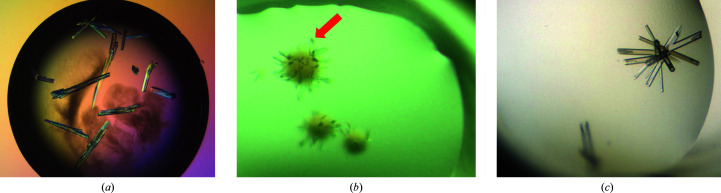
Crystals obtained for different variants of selenocysteine-containing human GPX4. (*a*) GPX4^WT^. The largest rods are about 200 × 40 × 40 µm in size. (*b*) GPX4^WT^ modified with ML162 (racemate). The crystal indicated by the red arrow is about 50 × 10 × 10 µm in size. (*c*) GPX4^C66S^ modified with enantiopure (*S*)-ML162 (the largest rod is about 200 × 30 × 30 µm in size).

**Figure 4:**
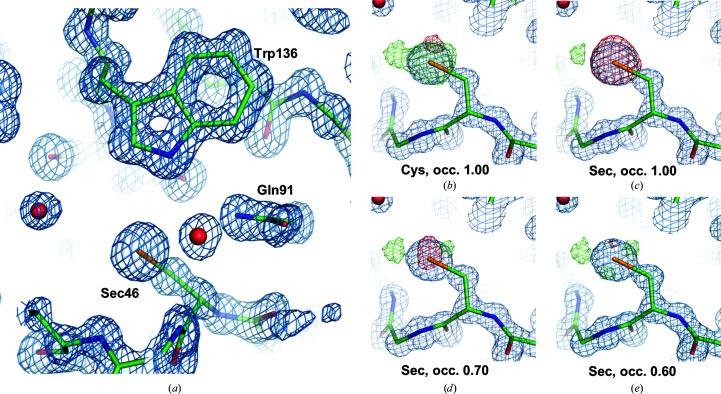
Electron-density maps for GPX4^WT^. (*a*) 2*mF*_o_ − *DF*_c_ map at the active site, contoured at 1.5σ, with the final GPX4^WT^ model shown in stick representation. Three residues of the catalytic tetrad are shown. The occupancy of the Se atom of Sec46 is 0.60. (*b*)–(*e*) show 2*mF*_o_ − *DF*_c_ maps (blue) contoured at 1.5σ after modeling residue 46 as cysteine (*b*) or as selenocysteine with different occupancies (*c*, *d*, *e*). Difference density (*mF*_o_ − *DF*_calc_) maps are contoured at 3σ (green) and −3σ (red), respectively.

The apo GPX4^WT^ protein crystallized under several conditions when subjected to a broad initial crystallization screen. One condition was further refined and produced rod-shaped crystals (Fig. 3 ▸ *a*) which diffracted to between 1.0 and 1.4 Å resolution using synchrotron radiation. Data-collection statistics are shown in Table 1 ▸. The structure was solved by molecular replacement. In the refined structure, the active-site selenocysteine residue Sec46 was clearly defined in the electron-density map, but the difference density maps showed a strong peak at the Se atom (Fig. 4 ▸). Modeling this residue either as cysteine (Fig. 4 ▸ *b*) or as selenocysteine with different occupancies for the Se atom (Figs. 4 ▸ *c* and 4 ▸ *d*) confirmed that selenocysteine was indeed present, albeit with reduced occupancy. Reducing the occupancy of the Se atom to 0.60 removed most of the difference density. The GPX4^WT^ sample used for crystallization featured 100% selenium incorporation, as confirmed by mass spectrometry (Fig. 2 ▸ *b*). The reduced occupancy of the Se atom in the structure may therefore be owing to radiation damage, aggravated by the long exposure time that was required to achieve sufficient completeness in space group *P*1. For selenomethionine side chains, the radiation damage caused by X-rays has been analyzed and breakage of the C—Se bond has been suggested to be the most likely mechanism (Holton, 2007 ▸). An equivalent mechanism would explain the partial loss of selenium observed here.

**Figure 5:**
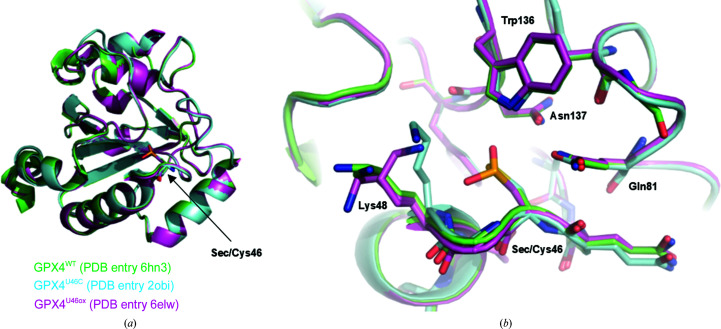
Comparison of wild-type GPX4 with previous GPX4 crystal structures. (*a*) Overall fold of GPX4^WT^ (PDB entry 6hn3), GPX4^U46C^ (PDB entry 2obi) and a mutant version of GPX4 in which position Sec46 was found to be oxidized to seleninic acid and all other cysteine residues were mutated to serine or alanine (PDB entry 6elw). Only the active-site residue Sec46/Cys46 is shown in stick representation for orientation. (*b*) View into the active site of GPX4. Residues lining the site around Sec46 are shown in stick representation and include the residues which form the catalytic tetrad (Sec46, Gln81, Trp136 and Asn137).

At the very high resolution obtained here, many side chains needed to be modeled in alternative conformations. Additionally, one of the histidine residues of the C-terminal hexahistidine tag could be built in the electron-density maps. The overall fold is in principle identical to the GPX4 mutant crystal structures reported previously. The r.m.s.d. over all C^α^ atoms is 1.12 Å for PDB entry 2obi (GPX4^U46C^; Scheerer *et al.*, 2007 ▸) and 0.73 Å for PDB entry 6elw (Sec46 introduced via mutation to cysteine and expression in cysteine-free medium supplemented with selenocysteine, with all other cysteine residues mutated to noncysteines; Borchert *et al.*, 2018 ▸). Fig. 5 ▸(*a*) shows a superimposition of the active site of GPX4^WT^ (PDB entry 6hn3, this study) with these two previous structures. Fig. 5 ▸(*b*) illustrates that the residues of the catalytic triad, Sec/Cys46, Gln81 and Trp136, superimpose very well. Only the side chain of Lys48 adopts a different rotamer, most likely as a result of different crystal packing and, in the case of PDB entry 6elw, a hydrogen bond between Lys48 and Sec46, which was found to be oxidized.

### 3.2. Mass-spectrometry-monitored generation, crystallization and structure determination of GPX4^WT^–ML162 (racemate)   

**Figure 6:**
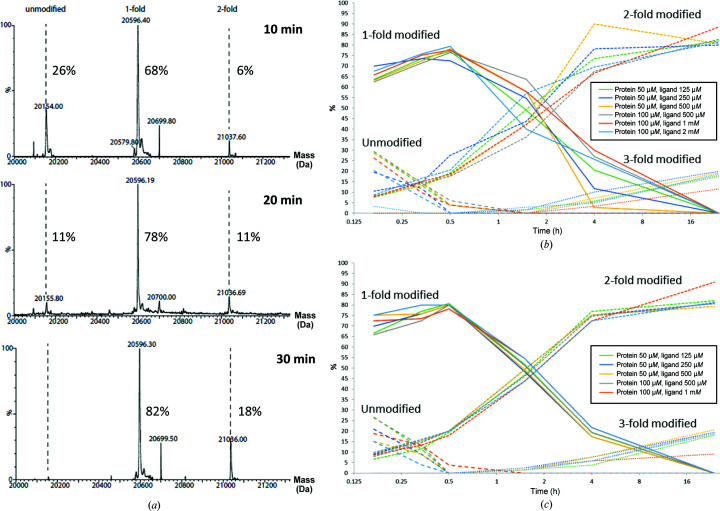
(*a*) Mass-spectrometric analyses of the covalent reaction of GPX4^WT^ with ML162 (racemate). Shown are deconvoluted mass spectra after 10, 20 and 30 min of incubating 50 µ*M* GPX4^WT^ with 125 µ*M* ML162 (racemate). (*b*, *c*) Mass-spectrometric analyses of the covalent reaction of GPX4^WT^ with (*b*) the pure (*S*)-enantiomer and (*c*) the pure (*R*)-enantiomer. A time-course analysis is shown of the observed GPX4 species (unmodified or onefold, twofold or threefold covalently modified with ML162) found at the given time points for the ligand:protein ratios given in the box.

**Figure 7:**
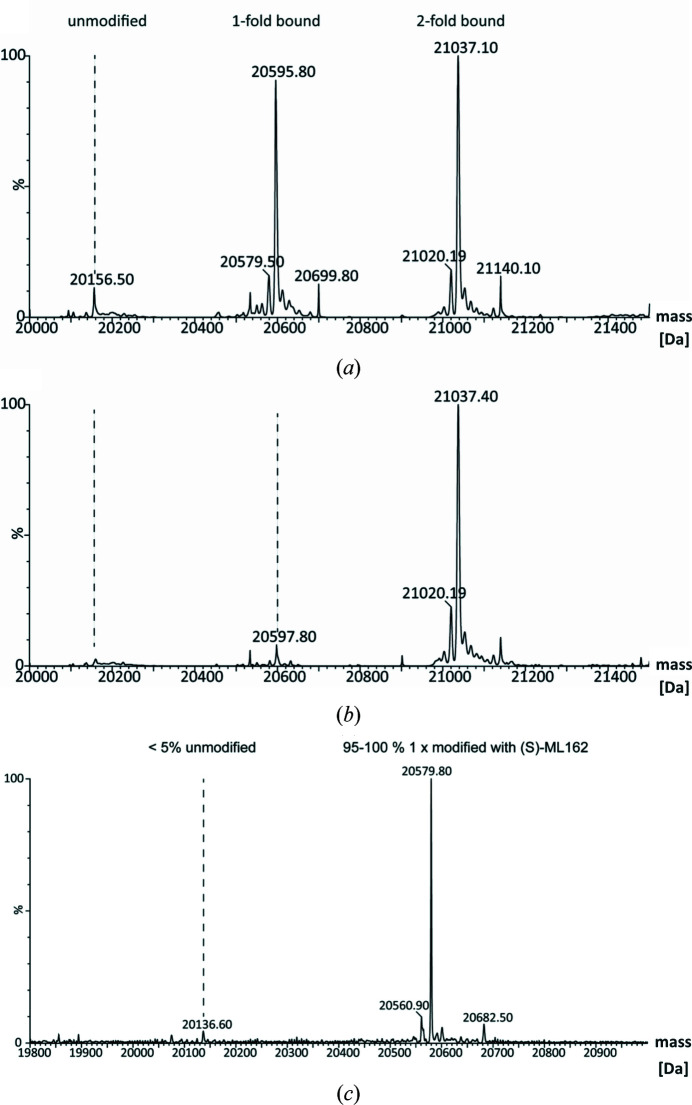
Mass-spectrometric analyses of the preparative-scale covalent reactions of different versions of GPX4 with ML162. (*a*) GPX4^WT^, outcome of approach 1 [50 µ*M* GPX4^WT^ incubated for 35 min with a fivefold molar excess of ML162 (racemate)], revealing a mixture of unmodified, singly modified and doubly modified GPX4. (*b*) GPX4^WT^, approach 2 [50 µ*M* GPX4^WT^ incubated for 4 h with a 50-fold molar excess of ML162 (racemate)], resulting in homogenously doubly modified protein. (*c*) Large-scale reaction of GPX4^C66S^ with (*S*)-ML162, indicating complete turnover of the protein to the singly modified state upon incubation of 50 µ*M* protein with 125 µ*M* ligand for 30 min.

With crystallizable GPX4^WT^ available, we set out to develop a protocol for the determination of co-crystal structures of GPX4 with covalent inhibitors. Initial efforts to generate crystals modified with the racemic mixture of ML162 via direct incubation of GPX4 followed by crystallization screening failed. We suspected that heterogeneous modification of GPX4 by ML162 might have impeded crystallization. Soaking of this crystal form failed as well, which may be caused by Arg12 of a symmetry-related molecule. This arginine residue reaches into the active site and probably blocks access by the inhibitor. We therefore decided to generate GPX4 homogenously modified with ML162 using a mass-spectrometry time-course experiment. Different GPX4:ML162 molar ratios were incubated at three different protein concentrations for 4 h and samples were taken at different time points. The reactions were stopped by the addition of TFA and the samples were analyzed by mass spectrometry (Fig. 6 ▸). Based on these results, two reaction conditions were selected for large-scale reproduction. 5 mg GPX4^WT^ (50 µ*M* final concentration) was incubated either for 35 min with a fivefold molar excess of ML162 (approach 1) or for 4 h with a 50-fold molar excess of ML162 (approach 2). The reaction mixtures were subjected to SEC (Superdex 75) to separate excess inhibitor and stop the reaction. The peak fractions were concentrated and subjected to a crystallization screen. Retrospective MS analysis of the samples used for crystallization revealed that for approach 1 the protein sample consisted of 50% of the mono-adduct and 50% of the double adduct (Fig. 7 ▸ *a*), while approach 2 yielded 100% double adduct (Fig. 7 ▸ *b*). Surprisingly, crystals were only obtained with the nonhomogeneously modified sample from approach 1. The crystals grew as very small rods (Fig. 3 ▸ *b*), belonged to space group *P*2_1_ and diffracted to a resolution of 2.3 Å. The structure could be solved using molecular replacement and contained two GPX4^WT^ molecules in the asymmetric unit. However, only one chain showed extra density, not only on the side chain of the catalytic Sec46 but also on Cys66, which was interpreted as covalently bound ML162. The reactive warhead of ML162, a chloracetamide group, is a relatively strongly reactive group. At the high inhibitor concentration used in this experiment, an additional adduct formation at the solvent-exposed residue Cys66 was therefore not fully surprising. As the density for the ligand was too weak to place it unambiguously both at Sec46 and Cys66, and as density for the phenethylacetamide arm of ML162 was completely absent, work on this structure was terminated. However, we concluded that mutating the surface-exposed cysteine residue Cys66 to serine may help to produce homogenously mono-modified GPX4 and thus may increase the likelihood of obtaining well diffracting crystals.

### 3.3. Crystal structure of GPX4^C66S^ in complex with (*S*)-ML162   

**Figure 8:**
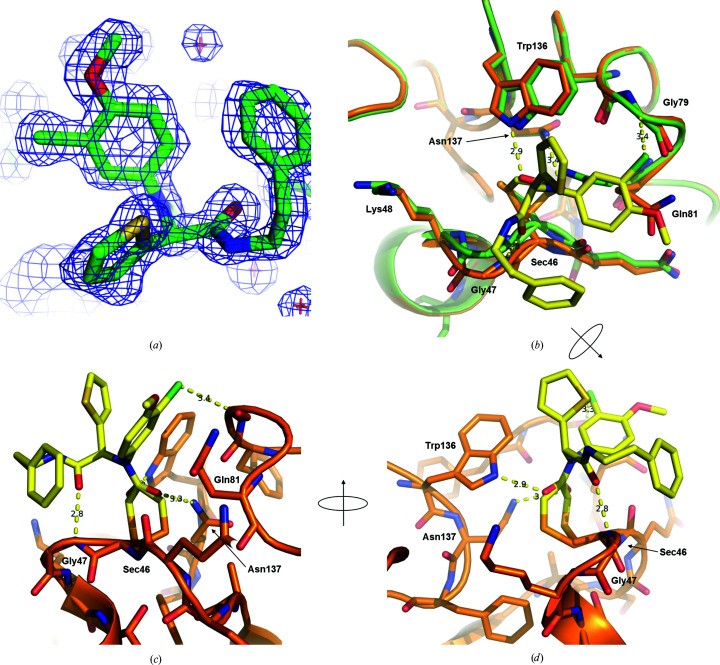
Crystal structure of GPX4^C66S^ in complex with covalently bound (*S*)-ML162. (*a*) 2*mF*_o_ − *DF*_c_ map contoured at 1.0σ showing the bound inhibitor. (*b*) Superimposition of the active sites of GPX4^WT^ (C atoms in green) and of the GPX4^C66S^–ML162 complex (C atoms in orange).

In order to obtain a better resolved co-complex structure with ML162, we first repeated the MS-monitored time-course experiment with GPX4^WT^, but now using the two pure enantiomers of ML162. This revealed very similar binding behavior for both enantiomers (Figs. 6 ▸ *b* and 6 ▸ *c*), but also still twofold and even threefold modification of GPX4^WT^ for both enantiomers. Based on signs of a more concentration-dependent reaction for one of the two enantiomers in the MS time course, we selected this enantiomer for upscaling the covalent reaction. To prevent the heterogenous modification observed both with the racemic mixture of ML162 and with the pure enantio­mers, we produced a mutant form of GPX4 in which the second reactive cysteine, Cys66, was mutated to a serine (termed GPX4^C66S^). 50 µ*M* GPX4^C66S^ was incubated with 125 µ*M* ML162 for 30 min and the reaction was stopped by gel filtration. Here, mass-spectrometric analysis showed >95% onefold modification, with less than 5% free GPX4 and no double adduct (Fig. 7 ▸ *c*). Crystals grew within one day (Fig. 3 ▸ *c*) and a data set was collected to 1.5 Å resolution on a rotating-anode source. The structure was solved by molecular replacement. The crystal belonged to space group *P*2_1_2_1_2_1_, with one GPX4^C66S^ molecule per asymmetric unit. The final data-collection and refinement statistics are summarized in Tables 1 ▸ and 2 ▸. Clear difference density allowed the building of a complete inhibitor ML162 covalently linked to Sec46. The density map unambiguously revealed that the stereoisomer used in this experiment was the (*S*)-enantiomer (Fig. 8 ▸ *a*).

### 3.4. Binding mode of (*S*)-ML162   

Superimposition with the apo GPX4^WT^ structure (Fig. 8 ▸ *b*) revealed that in the inhibitor co-complex the loop containing Sec46 has moved about 1 Å away from Trp136. As expected from mass spectrometry, the Cl atom of the inhibitor is lost and the acetamide moiety forms a covalent bond to the Se atom of Sec46. Interestingly, the Se atom and the covalently linked carbonyl group of the acetamide moiety of ML162 adopt two alternative conformations whereby the O atom forms hydrogen bonds to either the indole N atom of Trp136 (2.9 Å) or the side-chain N atom of Asn137 (3.4 Å; Figs. 8 ▸ *c* and 8 ▸ *d*). The side chain of Gln81 adopts a new conformation and rotates away from Sec46, which further enlarges the otherwise very small active site. This also enables a π–π stacking interaction between the methoxychlorophenyl ring of the inhibitor and the amide moiety of Gln81, and a weak hydrogen bond (3.4 Å) between the Cl atom and the C^α^ atom of Gly79 (Fig. 8 ▸ *c*). The O atom of the carbonyl group directly adjacent to the thiophene ring forms a direct hydrogen bond (2.8 Å) to the backbone amide N atom of Gly47 (Fig. 8 ▸ *c*). Surprisingly, the thiophene group of the inhibitor does not engage in any interactions and instead is directed towards the solvent. By interacting with Sec46, Gln81, Trp136 and Asn137, the inhibitor targets all of the residues of the catalytic tetrad of GPX4 and thus fully blocks the active site. It also achieves these crucial interactions with its chloroacetamide and methoxychlorophenyl moieties alone, whereas the adjacent stereocenter does not contribute any further interaction via its thiophene ring, and only one additional hydrogen bond (to Gly47) is formed via its phenethylacetamide group. Overall, these observations are consistent with the structure–activity relationship (SAR) reported by Weïwer and coworkers for the effects of ML162 and a series of close derivatives in cellular GPX4 activity assays (Weïwer *et al.*, 2012 ▸). Weïwer and coworkers did not observe any significant differences for the effects of the two pure enantiomers, which is consistent with the lack of crucial interactions of the stereocenter observed here. It is, however, worth noticing that a flip of the stereocenter could enable the phenethylacetamide moiety to insert into a groove on the surface of GPX4 located between Trp136 and Lys48 (Figs. 8 ▸ *b* and 9 ▸ *a*). Owing to a different side-chain rotamer of Lys48, this surface groove is not present in the crystal structure of apo GPX4^U46C^ (Scheerer *et al.*, 2007 ▸; Fig. 5 ▸). A crystal structure of GPX4 in complex with (*R*)-ML162 could verify this hypothesis, and work towards this aim is in progress.

**Figure 9:**
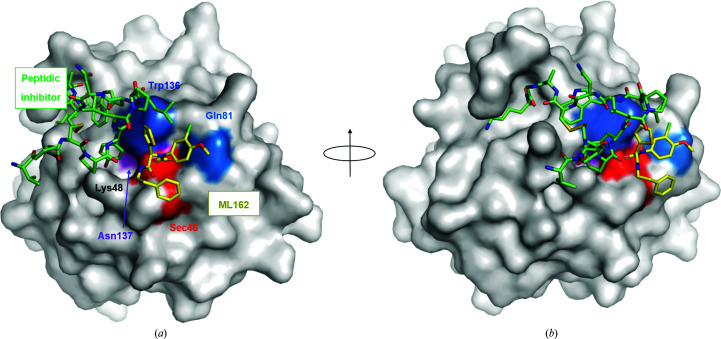
Comparison with the binding site of a known peptide inhibitor. The structure of GPX4^C66S^–(*S*)-ML162 is shown superimposed onto that of GPX4^U46C^ bound to a peptidic inhibitor (PDB entry 5h5s). For clarity only the peptide is shown for the latter, depicted with green C atoms. GPX4 is shown in surface representation, with the inhibitor (*S*)-ML162 with yellow C atoms, the surface of Sec46 colored red and the remaining residues of the catalytic triad in blue (Gln81), dark blue (Trp136) and purple (Asn137). (*a*) and (*b*) show two different orientations.

Sakamoto and coworkers reported the only co-crystal structure to date with an inhibitor binding close to the active site of GPX4 (PDB entry 5h5s; Sakamoto *et al.*, 2017 ▸). This cyclic peptide inhibitor extends over a larger part of the surface of GPX4 and binds immediately adjacent to Sec46, but does not form a covalent bond to it. Superimposition of this peptide complex with the (*S*)-ML162 co-crystal structure (Fig. 9 ▸) shows that the two inhibitors do not overlap at all. This may open the path towards the synthesis of hybrids where ML162 could be extended into adjacent subpockets seen in the peptide co-complex. Notably, Lys48 adopts a similar rotamer conformation as observed in our ML162 co-complex, the groove between Lys48 and Trp136 is open and the peptide inhibitor inserts a tyrosine residue into it. Covalent GPX4 inhibitors targeting Sec47 may in general also inhibit other selenocysteine-containing enzymes in the human proteome. Extending a covalent GPX4 inhibitor into subpockets adjacent to Sec47, such as the pocket between Lys48 and Trp136 described here, may provide a path forward towards more selective GPX4 inhibitors with fewer side effects.

## 4. Conclusions   

Biochemical and structural studies of GPX4 and its inhibitors have been hindered by the lack of an efficient source of wild-type GPX4 protein (Borchert *et al.*, 2018 ▸; Kernstock & Girotti, 2008 ▸; Yang *et al.*, 2016 ▸). The recombinant expression of mammalian selenoproteins in bacterial protein-production systems is often difficult owing to the low efficiency of selenocysteine incorporation (Han *et al.*, 2013 ▸; Scheerer *et al.*, 2007 ▸; Thyer *et al.*, 2015 ▸). Historically, structural studies of GPX4 have only been possible using U46C and U46G active-site mutants (Janowski *et al.*, 2016 ▸; Sakamoto *et al.*, 2017 ▸). The structure of a selenocysteine-containing GPX4 has recently been reported (Borchert *et al.*, 2018 ▸), but to date structural studies of covalent GPX4 inhibitors have not been successful (Yang *et al.*, 2016 ▸). Co-crystal structures of GPX4^U46C^ in complex with reversible peptide binders have been reported, but their relevance remains unknown given that these compounds are unable to inhibit cellular GPX4 (Sakamoto *et al.*, 2017 ▸).

In this manuscript, we report the first crystallization and structural determination of true wild-type GPX4 protein, as well as the first complex of GPX4 with a covalent small-molecule inhibitor. This was achieved by the co-expression of GPX4 with SECIS-binding protein 2 (SBP2) in HEK293-6E cells (Eaton *et al.*, 2020 ▸), which allowed the generation of true wild-type GPX4 and GPX4^C66S^ proteins. The apo wild-type GPX4 structure described here is very similar to previously reported mutant GPX4 structures (Borchert *et al.*, 2018 ▸; Janowski *et al.*, 2016 ▸; Scheerer *et al.*, 2007 ▸).

In a recently reported crystal structure of a mutant form of GPX4 which features a Sec46 residue, but in which all other cysteine residues have been mutated to noncysteine residues, the selenocysteine residue was oxidized and was modeled as seleninic acid (SeO_2_H; Borchert *et al.*, 2018 ▸). In the wild-type apo GPX4 structure reported here, there is only very weak difference density on the Se atom, which did not justify modeling it as an oxidized form. This difference in oxidation may be related to the tenfold higher concentration of the reducing reagent TCEP used in our study.

Generation of GPX4 crystals in complex with the GPX4 inhibitor ML162 was initially complicated by heterogeneous modification of GPX4 by ML162, including partial covalent modification of the surface-exposed cysteine residue Cys66 at high concentrations of ML162. Mutation of Cys66 to serine, along with optimization of binding reaction conditions and the use of enantiomerically pure (*S*)-ML162, enabled the determination of a co-crystal structure at 1.54 Å resolution. (*S*)-ML162 is fully defined in the electron density and is covalently linked to Sec46 (Fig. 6 ▸). In the absence of a deep binding pocket within the active site of GPX4, it occupies a shallow surface pocket at Sec46 where, in addition to the covalent bond to Sec46, it also forms interactions with the three other residues of the catalytical tetrad (Gln81, Trp136 and Asn137).

Our results provide the first insight into the structural basis of GPX4 inhibition by chloroacetamide inhibitors. ML162 occupies a different surface area of GPX4 to the inhibitory peptide described by Sakamoto and coworkers, which binds closest to the catalytic site of GPX4 (Sakamoto *et al.*, 2017 ▸). The lack of a well defined deeper binding pocket in the ML162 co-crystal structure reaffirms the difficulties associated with targeting GPX4 with small molecules. Based on these observations, it is unsurprising that currently known inhibitors target GPX4 through a covalent mechanism of action. However, the conformational changes observed upon the binding of (*S*)-ML162, both of active-site residues such as the rotamer changes of Gln81 and Lys48 and of the loop carrying Sec46, together with the observed flexibility of Lys48, indicate that the active site of GPX4 shows some plasticity and may adjust to a suitable inhibitor to result in more optimal binding and higher potency as well as higher selectivity. The GPX4 expression and co-crystallization strategy described here will facilitate the structural characterization of other GPX4 binders to support the rational development of inhibitors with improved drug-like properties.

## Supplementary Material

PDB reference: human GPX4, complex with covalent inhibitor ML162 (*S*-enantiomer), 6hkq

PDB reference: wild-type form with Sec46, 6hn3

## Figures and Tables

**Table 1:** Data collection and processing Values in parentheses are for the outer shell.

Data set	Apo GPX4^WT^	GPX4^C66S^–(*S*)-ML162
PDB code	6hn3	6hkq
Diffraction source	Beamline 14.1, HZB	Rigaku MicroMax-007 HF
Wavelength (Å)	0.9184	1.5418
Temperature (K)	100	100
Detector	PILATUS3 6M	PILATUS 200K
Crystal-to-detector distance (mm)	149	60
Rotation range per image (°)	0.1	0.1
Total rotation range (°)	2 × 180	155.6
Space group	*P*1	*P*2_1_2_1_2_1_
*a*, *b*, *c* (Å)	32.8, 35.2, 37.8	32.7, 57.3, 81.3
α, β, γ (°)	103.2, 112.3, 91.8	90.0, 90.0, 90.0
Mosaicity (°)	0.122	0.679
Resolution range (Å)	33.97–1.01 (1.03–1.01)	46.81–1.54 (1.59–1.54)
No. of unique reflections	79169	20009
Completeness (%)	94.7 (87.6)	86.6 (72.1)†
Multiplicity	1.8 (1.6)	10.5 (6.7)
〈*I*/σ(*I*)〉	14.6 (1.8)‡	20.2 (2.4)
CC_1/2_ (outer shell)	0.925	0.808
*R*_merge_	0.04 (0.34)	0.10 (0.90)
*R*_r.i.m._	n.d.	0.11 (0.97)
Overall *B* factor from Wilson plot (Å^2^)	12.3	12.3

† The completeness was <93% in the outer shell owing to a non-optimal data-collection strategy. The data completeness was above 93% for all shells up to 2.24 Å resolution.

‡ *I*/σ(*I*) in the outer shell is <2.0 Å, but data were added because CC_1/2_ was 0.925 in the outer shell (0.999 for the complete data set). *I*/σ(*I*) falls below 2.0 at a resolution of 1.06 Å.

**Table 2:** Structure solution and refinement Values in parentheses are for the outer shell.

Data set	Apo GPX4^WT^	GPX4^C66S^–(*S*)-ML162
PDB code	6hn3	6hkq
Resolution range (Å)	33.97–1.01 (1.03–1.01)	46.81–1.54 (1.58–1.54)
Completeness (%)	94.7	86.6
No. of reflections, working set	71219	19002
No. of reflections, test set	3749	951
Final *R*_cryst_	0.115 (0.250)	0.150 (0.226)
Final *R*_free_	0.137 (0.265)	0.184 (0.295)
No. of non-H atoms		
Protein	1470	1365
Chloride ion/ethanol	5	n/a
ML162	n/a	32
SO_4_^2−^ ion/ethylene glycol	n/a	21
Solvent	248	228
Total	1723	1646
R.m.s. deviations		
Bonds (Å)	0.016	0.013
Angles (°)	1.877	1.701
Average *B* factors (Å^2^)		
Protein	13.8	17.2
Chloride ion/ethanol	18.4	n/a
ML162	n/a	26.9
SO_4_^2−^ ion/ethylene glycol/DMSO	n/a	45.4
Water	27.0	30.6
Ramachandran plot†		
Most favored (%)	98.6	98.7
Allowed (%)	1.4	1.3
*MolProbity* score‡	1.72	1.09

† Ramachandran statistics calculated with *Coot*.

‡ *MolProbity* score calculated using the server at http://molprobity.biochem.duke.edu/.
